# A short-chain acyl-CoA synthetase that supports branched-chain fatty acid synthesis in *Staphylococcus aureus*

**DOI:** 10.1016/j.jbc.2023.103036

**Published:** 2023-02-16

**Authors:** Sarah G. Whaley, Matthew W. Frank, Charles O. Rock

**Affiliations:** Department of Infectious Diseases, St Jude Children’s Research Hospital, Memphis, Tennessee, USA

**Keywords:** *Staphylococcus aureus*, acyl-CoA synthetase, branched-chain ketoacid dehydrogenase, fatty acid synthesis, phospholipid, membrane, ACP, acyl carrier protein, BCAA, branched-chain amino acid, BCFA, branched-chain fatty acid, BHI, brain heart infusion, Bkd, branched-chain α-ketoacid dehydrogenase, Buk, butyrate kinase, FASII, type II fatty acid synthase, HSS, high strength silica, MbcS, methylbutyryl-CoA synthetase, MRM, multiple reaction monitoring, PG, Phosphatidylglycerol, Ptb, phosphotransbutyrylase, TBA, tributylamine, UFLC, Ultra Fast Liquid Chromatography, UPLC, ultra performance liquid chromotography

## Abstract

*Staphylococcus aureus* controls its membrane biophysical properties using branched-chain fatty acids (BCFAs). The branched-chain acyl-CoA precursors, utilized to initiate fatty acid synthesis, are derived from branched-chain ketoacid dehydrogenase (Bkd), a multiprotein complex that converts α-keto acids to their corresponding acyl-CoAs; however, Bkd KO strains still contain BCFAs. Here, we show that commonly used rich medias contain substantial concentrations of short-chain acids, like 2-methylbutyric and isobutyric acids, that are incorporated into membrane BCFAs. Bkd-deficient strains cannot grow in defined medium unless it is supplemented with either 2-methylbutyric or isobutyric acid. We performed a screen of candidate KO strains and identified the methylbutyryl-CoA synthetase (*mbcS* gene; *SAUSA300_2542*) as required for the incorporation of 2-methylbutyric and isobutyric acids into phosphatidylglycerol. Our mass tracing experiments show that isobutyric acid is converted to isobutyryl-CoA that flows into the even-chain acyl-acyl carrier protein intermediates in the type II fatty acid biosynthesis elongation cycle. Furthermore, purified MbcS is an ATP-dependent acyl-CoA synthetase that selectively catalyzes the activation of 2-methylbutyrate and isobutyrate. We found that butyrate and isovalerate are poor MbcS substrates and activity was not detected with acetate or short-chain dicarboxylic acids. Thus, MbcS functions to convert extracellular 2-methylbutyric and isobutyric acids to their respective acyl-CoAs that are used by 3-ketoacyl-ACP synthase III (FabH) to initiate BCFA biosynthesis.

Fatty acid synthesis is a central aspect of bacterial metabolism and is carried out by a series of reactions catalyzed by individual proteins known as the type II fatty acid synthase (FASII) (1). Fatty acids are building blocks for membrane phospholipids and the membrane biophysical properties are controlled by the composition of the acyl chains (2). Bacteria modulate fatty acid composition by either altering the saturated:unsaturated fatty acid ratio or the straight-chain:branched-chain fatty acid ratio. Unsaturated fatty acids are formed at a branch point within FASII in bacteria that synthesize straight-chain fatty acids (1, 2) but in organisms like *Staphylococcus aureus* the branched-chain is introduced at the FabH condensation step that initiates FASII (2, 3, 4) (Fig. 1). Membranes with 15 to 17 carbon branched-chain fatty acids (BCFA) have reduced lipid bilayer thickness and an increased bilayer fluidity compared to their straight-chain counterparts (5, 6). Fatty acids with *anteiso* branching are more effective at fluidizing the membrane than fatty acids with *iso* branching. An increase in the proportion of *anteiso* fatty acids as an adaptive response to reduced temperatures is especially important for the survival of cold tolerant pathogens like *Listeria monocytogenes* (7, 8, 9).

**Figure 1:**
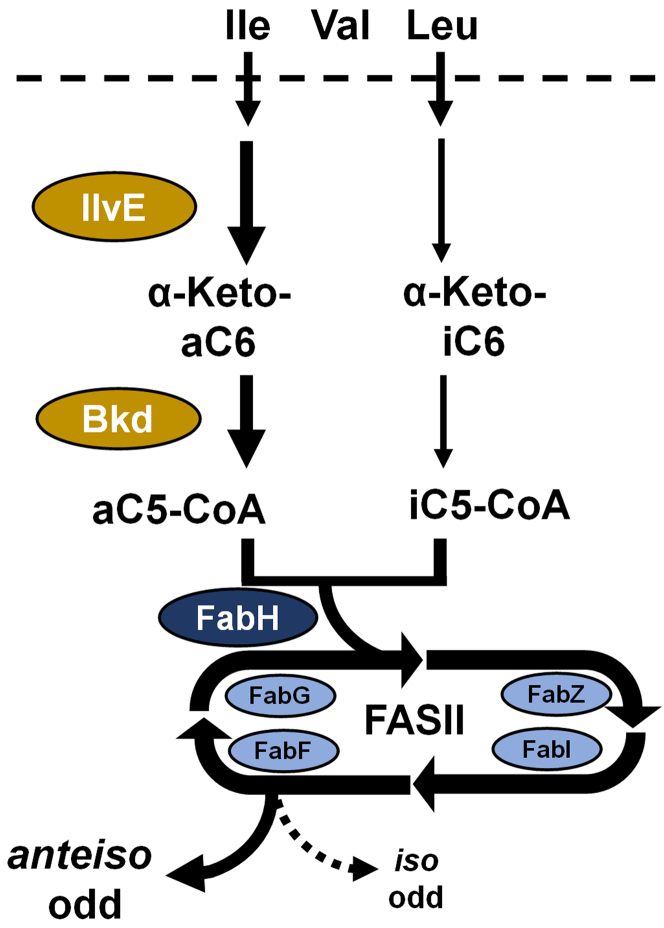
**Pathway for the utilization of extracellular branched-chain amino acids (BCAA) for branched-chain fatty acid (BCFA) biosynthesis in *S. aureus*.** Extracellular BCAA are transported into the cell by multiple BCAA transporters and the BCAA transaminase (IlvE) selectively converts Ile and Leu to their respective α-ketoacids. The branched-chain ketoacid dehydrogenase complex (Bkd) converts these α-ketoacids to their respective branched-chain acyl-CoAs. These acyl-CoAs are used by 3-ketoacyl-ACP synthase III (FabH) to initiate the bacterial fatty acid biosynthesis (FASII) elongation cycle to produce BCFA. Ile is the preferred substrate and Val is not a significant contributor to BCFA synthesis when Ile or Leu are present. Ile metabolism gives rise to *anteiso* odd-carbon number BCFA and Leu gives rise to *iso* odd-carbon BCFA. ACP, acyl carrier protein.

Extracellular branched-chain amino acids (BCAA; Ile, Leu, and Val) are transported into the cell by multiple BCAA transport proteins (10, 11). The first step in the utilization of BCAA for fatty acid synthesis in *S. aureus* is IlvE, a transaminase that converts these amino acids to their corresponding α-ketoacids (Fig. 1) (7, 12, 13). The α-ketoacids are then converted to acyl-CoAs by branched-chain α-ketoacid dehydrogenase (Bkd) (Fig. 1), an enzyme complex composed of four proteins encoded by the four-gene *Bkd* operon (14). It is known that isoleucine is the preferred amino acid for this pathway, followed by leucine, with valine being a poor precursor for fatty acids (15). It is recognized that *Bkd* KO strains in *S. aureus* still contain substantial amounts of BCFA (14). This means that there is a Bkd-independent alternate route to branched-chain acyl-CoA. One candidate for Bkd bypass was pyruvate dehydrogenase based on its ability to convert branched-chain α-ketoacids to acyl-CoAs *in vitro* (16); however, pyruvate dehydrogenase *Bkd* double KO strains still contain BCFA (17). A branched-chain α-ketoacid decarboxylase, produces an aldehyde from α-ketoacids (18) and is a potential *de novo* bypass route. A branched-chain α-ketoacid decarboxylase pathway would also require two additional unidentified enzymes to convert the aldehyde to the acid and activate the short-chain acid to its acyl-CoA. CidC, a pyruvate:menaquinone oxidoreductase (19) is another candidate but this enzyme uses oxygen and also requires the activation of the resulting short-chain acid. Finally, it is known that extracellular short-chain acids, like 2-methylbutyrate (aC5), are incorporated into BCFA in *Bacillus subtilis* and *S. aureus* (20, 21, 22, 23), suggesting the presence of an activation pathway to form short-chain acyl-CoAs. *L. monocytogenes* has a butyrate kinase/phosphotransbutyrylase (Buk/Ptb) system that is responsible for the two-step activation of extracellular aC5 to aC5-CoA (24, 25). The genes encoding Buk and Ptb are located adjacent to the *Bkd* operon in *Listeria* and *Bacillus*; however, these genes are not present in the *S. aureus* genome.

Here, we show that *S. aureus* strains that lack a functional Bkd complex produce BCFA by salvaging short-chain acids present in the medium. Complex growth media commonly used to grow *S. aureus* strains contain substantial amounts of aC5, isobutyrate (iC4) and other short-chain acids. Bkd-deficient strains require exogenous aC5 or iC4 to grow in defined media. A focused mutant screen identified the *SAUSA300_2542* gene as necessary and sufficient for the incorporation of extracellular aC5 and iC4. The purified protein is a methylbutyryl-CoA synthetase (MbcS) that uses ATP to activate the short-chain acids to their respective acyl-CoA derivatives. Purified MbcS selectively activates aC5 and iC4 consistent with the selectivity in the incorporation of short-chain acids by *S. aureus*. Thus, there is not a *de novo* biosynthetic bypass for Bkd but rather MbcS functions as an aC5/iC4-CoA synthetase that scavenges these exogenous short-chain acids from the environment for use in membrane biogenesis.

## Results

### Fatty acid composition and growth of Bkd-deficient strains

**Figure 2:**
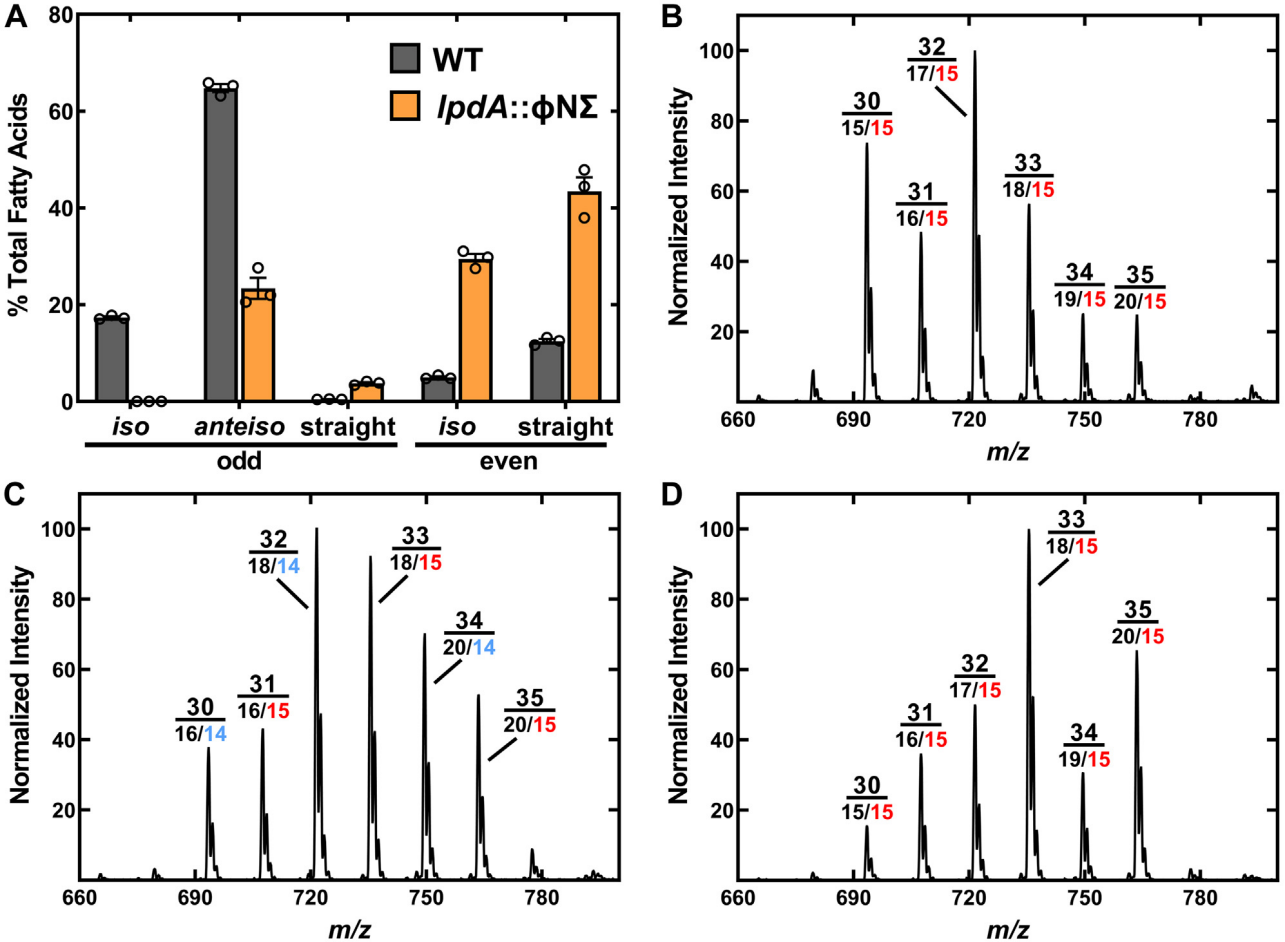
**Lipid composition of Bkd-deficient strains grown in LB.***A*, strains JE2 (WT) and NE1896 (*lpdA*::φNΣ) were grown in LB and the fatty acid compositions were determined by GC. Fatty acids are grouped into three categories of odd-carbon and two categories of even-carbon acyl chains. *B*, the PG molecular species composition of WT strain JE2 grown in LB. *C*, PG molecular species of strain NE1896 (*lpdA*::φNΣ) grown in LB. *D*, PG molecular species of strain NE1896 (*lpdA*::φNΣ) grown in LB supplemented with 1 mM 2-methylbutyrate (aC5). Peaks in the PG mass spectra are labeled with the total number of fatty acid carbons above the major 1-position/2-position acyl group combination found in the peak. The *anteiso* 15-carbon acyl chain is *red* and the *iso* 14-carbon acyl chain is cyan. PG, phosphatidylglycerol.

Phosphatidylglycerol (PG) is the most abundant membrane lipid in *S. aureus*. We used strain NE1896 (*lpdA*::φNΣ) containing a mariner-based *bursa aurealis* transposon (φNΣ) insertion in the leading gene of the four-gene *Bkd* operon (Fig. S1*A*) (26) to inactivate Bkd function. Strain NE1896 (*lpdA*::φNΣ) was grown in LB and the fatty acid composition determined (Fig. 2*A* and Table S1). Compared to WT strain JE2, there was an increase in straight and *iso* even-chain fatty acids, a decrease in *anteiso* odd-chain fatty acids, and *iso* odd-chain became rare in strain NE1896 (*lpdA*::φNΣ). These changes in fatty acid composition are similar to the differences between other WT and Bkd-deficient strain pairs that have been reported (14, 17, 27, 28). Although there is an overall decrease in total BCFA, BCFA remain quite abundant pointing to a Bkd-independent pathway to these BCFA. We obtained the same shift in fatty acid composition using strain NE1829 (*bkdA2*::φNΣ) as the Bkd-deficient strain (Fig. S1*B*). The PG molecular species composition of *S. aureus* strain JE2 grown in LB was essentially the same as observed in other WT strains grown in complex-rich medias (14, 29, 30). Almost all PG molecular species contain *anteiso*-15:0 (a15) in the 2-position and the major PG species has 32 carbons and consists of a17/a15-PG (1-position/2-position-PG) (Fig. 2*B*). The PG molecular species composition of the *lpdA*::φNΣ strain was quite different from the WT parent (Fig. 2*C*).

MS was used to determine the positional acyl chain distribution on the glycerol backbone of PG. The 2-position fatty acid is ∼2-fold more abundant than the 1-position acyl chain when a PG peak is fragmented (15, 30, 31, 32, 33), allowing the positional assignment of acyl chains in the peak (Fig. S2) based on the knowledge of the total fatty acid composition (Fig. 2*A* and Table S1). The 32-PG peak remained abundant in the Bkd-deficient strain but the primary molecular species was 18/i14-PG rather than a17/a15-PG found in the WT parent (Fig. S2*A*). The substitution of i14 for a15 in the 2-position was observed in all the even carbon PG peaks (30, 32, 34) (Fig. 2*C* and S2*A*). In contrast, the odd-carbon PG peaks (31, 33, 35) consisted of an even-chain fatty acid in the 1-position combined with a15 in the 2-position in both the WT and Bkd-deficient strains (Fig. 2*C* and S2*B*). The same replacement pattern was observed in strain NE1829 (*bkdA2*::φNΣ) (Fig. S1*C*). These data show that in the absence of Bkd activity, a15 in the 2-position is replaced by i14 chains in the even-carbon PG molecular species series and a15 remains the major 2-position acyl chain in the odd-carbon PG. The *iso* odd-chain fatty acids were not detected in the Bkd-deficient strains (Fig. 2*A*).

The addition of 1 mM aC5 to the media resulted in a shift in the fatty acid composition (Table S1). The addition of aC5 increased *anteiso* BCFA and decreased *iso* even-carbon BCFA, while straight even-number fatty acids remained abundant. The change in fatty acid composition was reflected in the PG molecular species composition of strain NE1896 (*lpdA*::φNΣ) (Fig. 2*D*). The notable changes occurred in the even carbon peaks. The major species in the 30-PG peak changed from 16/i14-PG to a15/a15-PG, the 32-PG peak changed from 18/i14-PG to a17/a15-PG, and the 34-PG peak changed from 20/i14-PG to a19/a15-PG (Fig. 2*D* and S2*A*) in the presence of aC5. The odd-numbered PG peaks were composed of even-chain fatty acid paired with a15 in the absence of the aC5 supplement and remained the same in the presence of aC5 (Fig. 2*D* and S2*B*). These same changes in PG molecular species composition following aC5 addition to the media were observed when we used strain NE1829 (*bkdA2*::φNΣ) to inactivate Bkd (Fig. S1*D*). These data show that extracellular aC5 was incorporated into *anteiso*-BCFA resulting in the reduced representation of *iso*-BCFA in membrane PG.

### Entry of extracellular aC5 into the FASII pathway

**Figure 3:**
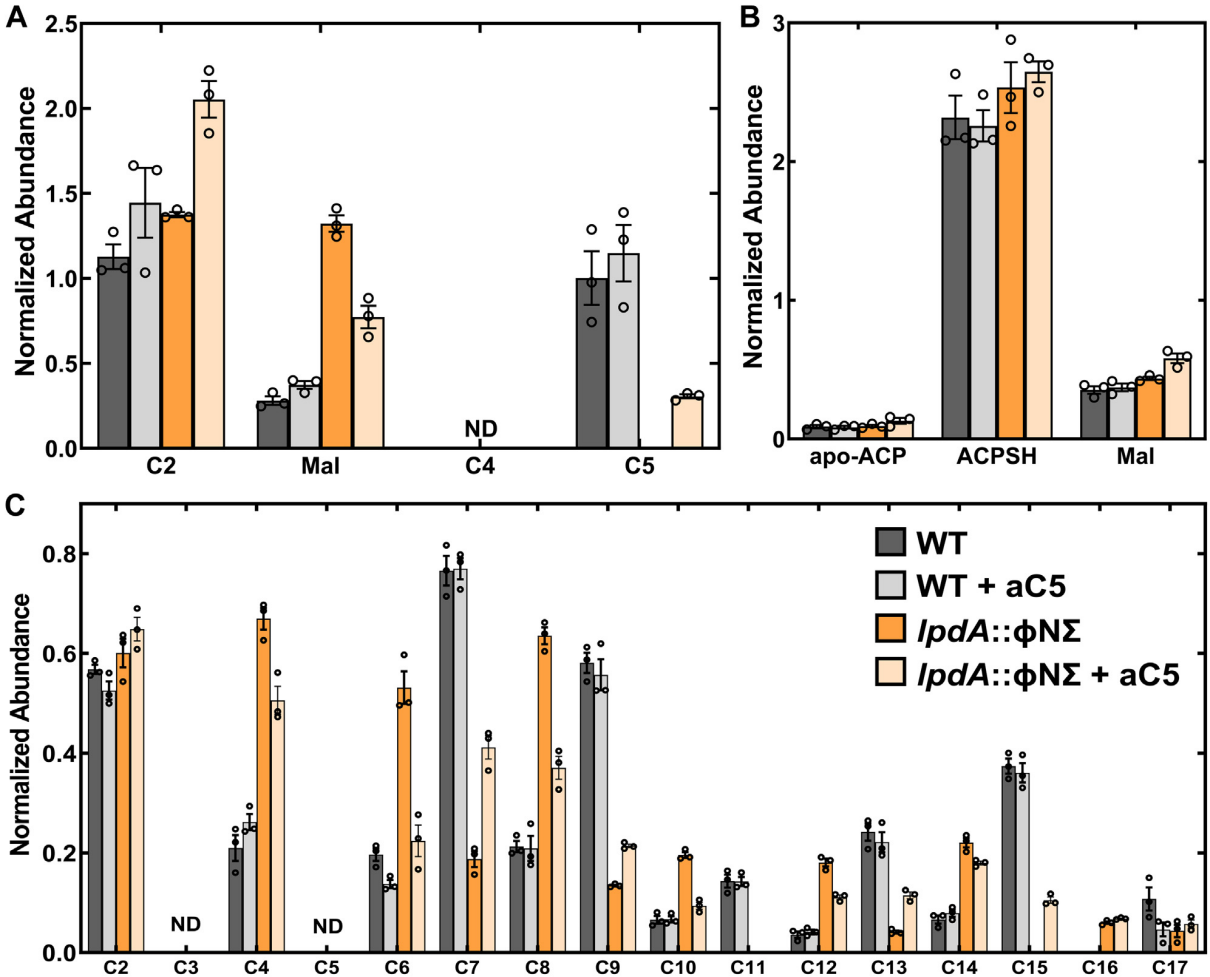
**Impact of aC5 supplementation on the acyl-CoA and acyl-ACP pool compositions in strain NE1896 (*lpdA*::φNΣ).** Strains were grown in LB or LB supplemented with 1 mM aC5. *A*, acyl-CoA pool compositions of strains JE2 (WT) and NE1896 (*lpdA*::φNΣ) in the presence and absence of aC5. Acyl-CoAs were measured using LC-MS/MS with [^13^C_2_]acetyl-CoA as the internal normalization standard. *B*, the amounts of apo-ACP, ACPSH, and malonyl-ACP (Mal) in strains JE2 (WT) and NE1896 (*lpdA*::φNΣ) in the presence and absence of aC5. ACP species were measured using LC-MS/MS using [^13^C_2_]acetyl-ACP as the internal standard to calculate normalized abundance. *C*, the FASII cycle intermediate acyl-ACP pool composition in strains JE2 (WT) and NE1896 (*lpdA*::φNΣ) strains in the presence and absence of aC5. *Inset*, the color key for all three panels. ACP, acyl carrier protein; FASII, type II fatty acid synthase.

Although FabH prefers aC5-CoA (3, 4, 13), the *S. aureus* fatty acid composition is determined predominately by the composition of acyl-CoA primers available to FabH (15). The *anteiso* odd-chain BCFA arise from Ile *via* 2-methylbutyryl-CoA (aC5-CoA), the *iso* odd-carbon BCFA arise from Leu *via* isovaleryl-CoA (iC5-CoA), the *iso* even-carbon fatty acids arise from Val *via* isobutyryl-CoA (iC4-CoA), and the straight, even-carbon fatty acids arise from acetyl-CoA (Fig. 1). We analyzed the acyl-CoA precursor pool compositions using LC-MS/MS to determine how aC5 was entering the biosynthetic pathway. The WT strain JE2 primarily contained acetyl-CoA, malonyl-CoA, and C5-CoA (aC5- + iC5-CoA) precursors that are used by FabH to initiate BCFA biosynthesis (15) and the amounts of these intermediates were not altered in strain JE2 by the addition of aC5 to the media (Fig. 3*A*). Strain NE1896 (*lpdA*::ΦNΣ) had higher levels of malonyl-CoA and the C5-CoA pool was below detection consistent with the Bkd deficiency (Fig. 3*A*). The addition of aC5 to the *lpdA*::φNΣ strain reduced the malonyl-CoA concentration and partially restored the C5-CoA pool (Fig. 3*A*). These data are consistent with exogenous aC5 being activated to aC5-CoA, which is used by FabH to produce *anteiso*-odd BCFA.

The FASII acyl-acyl carrier protein (ACP) pool compositions were also determined by LC-MS/MS. There were no differences in apo-ACP, ACPSH, or malonyl-ACP in either the WT or *lpdA*::φNΣ strains grown in LB either with or without an aC5 supplement (Fig. 3*B*). However, the composition of the acyl-ACP intermediates in the FASII elongation cycle were significantly altered in the *lpdA*::φNΣ strain (Fig. 3*C*). The even numbered acyl-ACP intermediates all increased in the absence of Bkd activity beginning with C4-ACP indicating elevated FabH initiation using acetyl-CoA. The odd-chain acyl-ACP intermediates decreased in the *lpdA*::φNΣ strain (Fig. 3*C*) reflecting the lack of available aC5- or iC5-CoA (Fig. 3*A*) for FabH to initiate BCFA biosynthesis. These differences in intermediate pool composition of FASII signal a switch from odd-to even-chain acyl-ACP FASII intermediates in the *lpdA*::φNΣ strain. The addition of aC5 to the media triggered the same changes in the amounts of even and odd-carbon 3-hydroxy-acyl-ACP (Fig. S3*A*) and trans-2-acyl-ACP (Fig. S3*B*). These data are consistent with the entry of extracellular aC5 into the FASII pathway by its conversion to aC5-CoA resulting in an increase in aC5-CoA utilization by FabH coupled with a decrease in initiation using acetyl-CoA and iC4-CoA when the cells are supplemented with extracellular aC5.

### Bkd-deficient strains are aC5/iC4 auxotrophs

**Figure 4:**
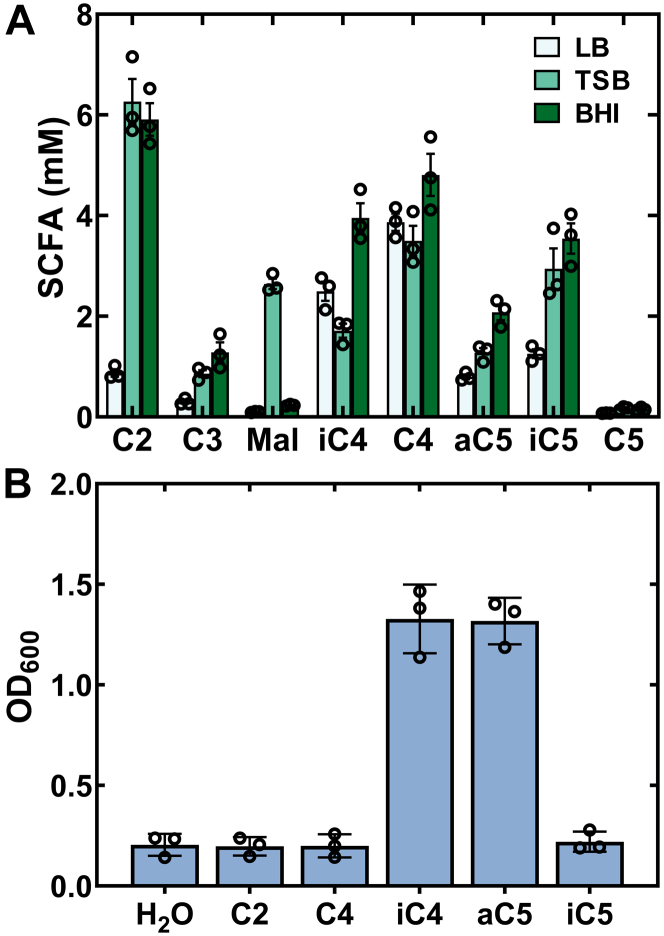
**Strain NE1896 (*lpdA*::φNΣ) is a short-chain fatty acid (SCFA) auxotroph.***A*, concentrations of short-chain fatty acids in complex-rich medias determined by LC-MS/MS of their picolinamide derivatives using [d9]isovaleric acid as the internal standard for quantification. LB, tryptic soy broth (TSB), and brain-heart infusion (BHI) broth. *B*, growth of strain NE1896 (*lpdA*::φNΣ) in defined media supplemented with 100 μM each of the indicated individual short-chain acids. Acetate (C2), butyrate (C4), isobutyrate (iC4), 2-methylbutyrate (aC5), and isovalerate (iC5).

These lipidomic and metabolic analyses suggested that complex, undefined media may contain unappreciated amounts of short-chain acids that the *lpdA*::φNΣ strain is utilizing to synthesize BCFA. Therefore, we analyzed three commonly used complex-rich medias for the presence of short-chain acids by LC-MS/MS (Fig. 4*A*). All three complex-rich medias [LB, tryptic soy broth, and brain heart infusion (BHI)broth] had significant concentrations of short-chain acids that if converted to their acyl-CoAs by cellular enzyme(s) could be utilized by FabH to initiate BCFA biosynthesis. We next studied the growth of strain NE1896 (*lpdA*::φNΣ) in a chemically defined growth media that supports the growth of WT *S. aureus* (15). The Bkd-deficient strain NE1896 (*lpdA*::φNΣ) failed to grow unless supplemented with a mixture of short-chain acids that were detected in the complex growth media (not shown). Testing each individual short-chain acid for their ability to support the growth of strain NE1896 (*lpdA*::φNΣ) showed that the growth of the Bkd-deficient strain in defined media required either aC5 or iC4 added as a supplement (Fig. 4*B*). The addition of acetate (C2), butyrate (C4), or isovalerate (iC5) to the medium did not support the growth of the Bkd-deficient strain. These data imply that the sole reason Bkd-deficient strains were able to grow in rich media was due to the presence of aC5 and iC4 in these formulations.

### Identification of the MbcS gene

The metabolomic analyses point to extracellular aC5 and iC4 as alternate sources of aC5-CoA and iC4-CoA that are used by FabH to initiate BCFA biosynthesis. We reasoned that in the absence of Buk/Ptb genes in *S. aureus*, that a gene encoding a short-chain acyl-CoA synthetase could account for aC5/iC4 activation to aC5/iC4-CoA and incorporation into BCFA *via* FabH. We obtained KO strains from the Nebraska transposon mutant library collection (26) in each of the four *S. aureus* genes annotated as acetyl/acyl-CoA synthetases. When Ile/Leu are removed from defined media, there is a distinct shift in the PG molecular species composition that arises from WT *S. aureus* using the *de novo* BCAA pathway, rather than the IlvE-dependent salvage pathway, to generate aC5-CoA and iC4-CoA for BCFA biosynthesis (15). The predominant PG molecular species in strain JE2 grown in defined media was 32:0-PG consisting of a17/a15-PG (Fig. S4*A*). The addition of extracellular aC5 did not significantly alter this PG molecular species distribution (not shown). In media lacking Ile and Leu, the PG molecular species were distinctly different (Fig. S4*B*), and we observed that aC5 supplementation restored the normal PG molecular species composition in *S. aureus* strain JE2 grown in defined media lacking Ile and Leu (Fig. S4*C*). This change was quantified by measuring the 32:35-PG peak ratio, which changes from 3.7 in defined media to 0.9 in the absence of Ile/Leu and back to 2.1 when an aC5 supplement was provided in the absence of Ile/Leu (Fig. S4). A dose-response experiment showed that 50 μM aC5 was sufficient to maximally alter the 32:35-PG ratio (Fig. S4*D*).

**Figure 5:**
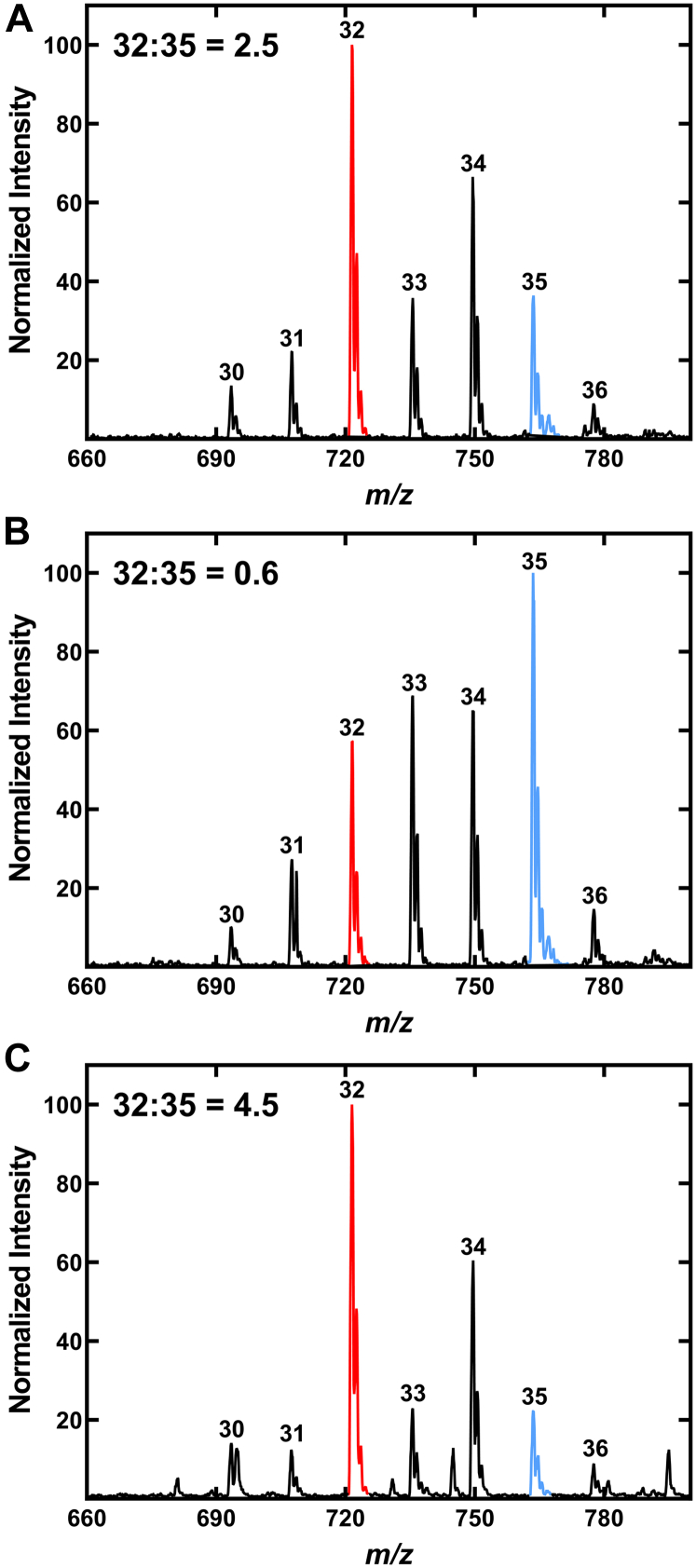
**Identification of the *mbcS* gene.** Strains were grown in defined media lacking Ile and Leu and supplemented with 50 μM aC5. *A*, aC5 supplementation corrects the change in PG molecular species phenotype of strain JE2 (WT) and gives a 32:35-PG ratio of 2.5. *B*, the PG molecular species of strain NE1036 (*mbcS*::φNΣ) remained unchanged by aC5 supplementation with a 32:35-PG ratio of 0.6. *C*, complementation of strain NE1036 (*mbcS*::φNΣ) with the MbcS expression plasmid pMbcS gives a 32:35-PG ratio of 4.5. 32-PG (*red*); 35-PG (*cyan*). MbcS, methylbutyryl-CoA synthetase; PG, phosphatidylglycerol.

The PG molecular species composition of the WT and four KO strains were determined in the presence and absence of 50 μM aC5 and the 32:35-PG ratio determined in each case (not shown). aC5 supplementation of strain JE2 increased the 32:35-PG ratio from less than 1 (Fig. S4*B*) to 2.5 (Fig. 5*A*). In contrast, the 32:35-PG ratio did not change with aC5 supplementation in strain NE1036 (Fig. 5*B*), which contains an inactivating insertion in the *SAUSA300_2542* gene predicted to encode an acetyl-CoA synthetase. These data indicate that *SAUSA300_2542* encodes a short-chain acyl-CoA synthetase capable of activating aC5 to aC5-CoA and we have named the gene *MbcS*. To determine if the *MbcS* gene was necessary and sufficient for aC5 incorporation into PG, the KO strain NE1036 (*mbcS*::φNΣ) was complemented with pMbcS, a plasmid expressing the *SAUSA300_2542* gene under control of a cadmium-inducible promoter (Tables S2 and S3). The plasmid-driven expression of MbcS in strain NE1036 (*MbcS*::φNΣ)/pMbcS showed the reversion of the strain to the WT pattern with a 32:35-PG ratio of 4.5 (Fig. 5*C*). These data show that MbcS is necessary and sufficient for the incorporation of extracellular aC5 into membrane phospholipids.

### Selectivity for short-chain acid incorporation

**Figure 6:**
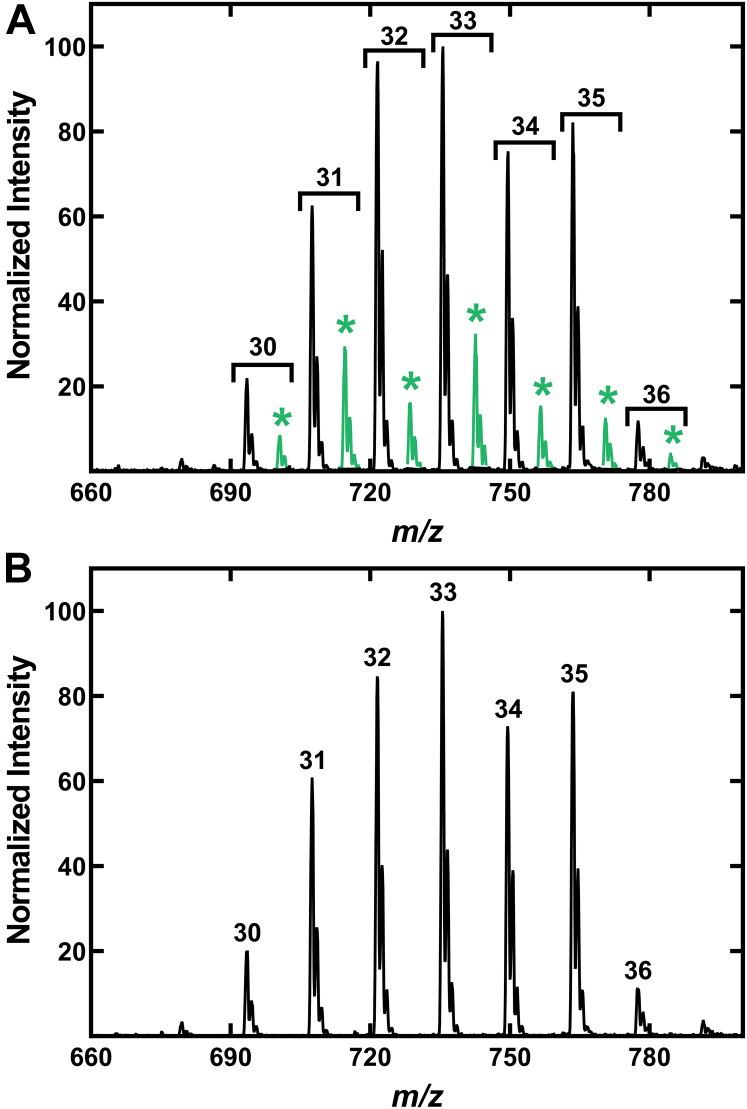
**Selectivity in the incorporation of extracellular short-chain acids by *S. aureus*.** Strain JE2 was grown in defined media lacking both Ile and Leu and supplemented with either 25 μM [d7]isobutyrate or [d9]isovalerate. *A*, PG molecular species of strain JE2 grown with [d7]isobutyrate. The +7 PG molecular species (∗) indicate the incorporation of [d7]isobutyrate into even-chain *iso* BCFA containing PG molecular species (highlighted in *green*). *B*, PG molecular species of strain JE2 grown in media containing [d9]isovalerate. Incorporation of [d9]isovalerate into +9 PG species was not detected. BCFA, branched-chain fatty acid; PG, phosphatidylglycerol.

**Figure 7:**
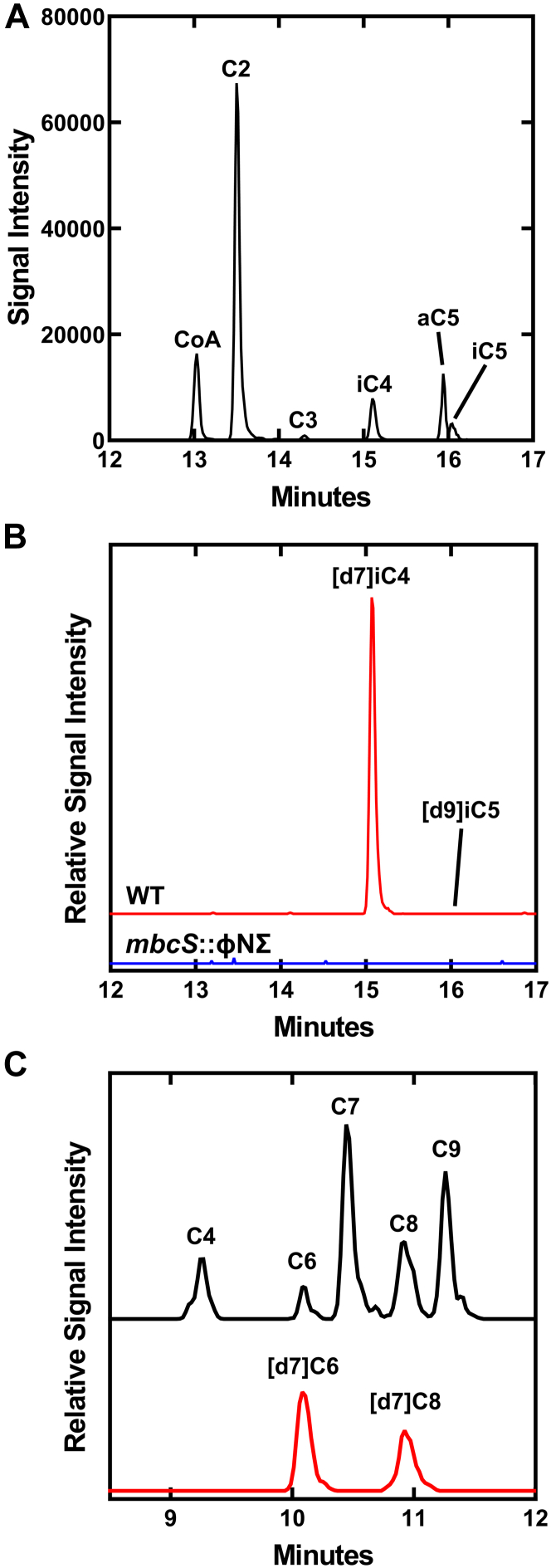
**Incorporation of [d7]isobutyrate into [d7]isobutyryl-CoA and its use in initiating the FASII elongation cycle.** Strain JE2 (WT) was grown in defined media lacking Ile and Leu supplemented with 50 μM each of [d7]isobutyrate and [d9]isovalerate. Cells were extracted and analyzed by LC-MS/MS for the appearance of mass-tagged acyl-CoA and acyl-ACP intermediates. *A*, acyl-CoA pool composition of strain JE2 using the normal mass MRMs for detection. *B*, the *red trace* shows the detection of (+7)iC4-CoA and (+9)iC5-CoA. Incorporation of [d7]isobutyrate into (+7)iC4-CoA was not detected in strain NE1036 (*mbcS*::φNΣ) (*blue trace*). *C*, incorporation of [d7]isobutyrate into the <C10 acyl-ACP FASII intermediates. The *black trace* shows the normal mass C4-C9 acyl-ACP pool composition of strain JE2. The red trace shows +7 mass tagged even-chain acyl-ACP species illustrating the incorporation of [d7]isobutyrate. The incorporation of a +9 mass tag into odd-chain acyl-ACPs from [d9]isovalerate was not detected. ACP, acyl carrier protein; FASII, type II fatty acid synthase; MRM, multiple reaction monitoring.

The incorporation of unlabeled aC5 was detected as described above; however, measurements of the incorporation of iC4 and iC5 required the use of mass-tagged [d7]isobutyrate and [d9]isovalerate to trace the entry of these acids into PG. Strain JE2 was grown in media lacking Ile and Leu and containing 50 μM each of [d7]isobutyrate and [d9]isovalerate to label the PG and intermediate pools. There was clear evidence for the activation of [d7]isobutyrate and its incorporation into PG based on the abundance of (+7)PG molecular species detected (Fig. 6*A*). In contrast, there was no evidence for the incorporation of [d9]isovalerate into PG (Fig. 6*B*). The intracellular acyl-CoA pool analysis of strain JE2 grown without Ile or Leu showed the presence of iC4- and aC5-CoA thioesters (Fig. 7*A*). Samples from cells labeled with [d7]isobutyrate plus [d9]isovalerate showed the formation of [d7]iC4-CoA from [d7]isobutyrate (Fig. 7*B*). The (+9)iC5-CoA that would be derived from [d9]isovalerate was not detected. The incorporation of neither [d7]isobutyrate nor [d9]isovalerate into acyl-CoAs was detected in strain NE1036 (*mbcS*::φNΣ) (Fig. 7*B*). We also examined the incorporation of the mass-tagged acids into the early (<C10) acyl-ACP intermediates in FASII elongation cycle. C4-C9 acyl-ACP with normal masses were detected but [d7]isobutyrate was only incorporated into the C6- and C8-ACP based on the appearance of these two (+7)acyl-ACP intermediates (Fig. 7*C*). The fact that mass-tagged C4-ACP was not detected means that (+7)iC4-CoA is condensed with malonyl-ACP by FabH to give rise to (+7)C6-ACP as the first labeled acyl-ACP intermediate. The C4-ACP arose from the FabH condensation of acetyl-CoA with malonyl-ACP. Odd-carbon (+9)acyl-ACPs were not detected consistent with the absence of [d9]isovalerate labeling of the acyl-CoA pool (Fig. 7*B*). These data show that extracellular iC4 is a precursor to BCFA *via* its activation to iC4-CoA by the *mbcS* gene product, which is then used by FabH to initiate FASII leading to its incorporation into even-carbon acyl-ACP. Extracellular iC5 was not efficiently incorporated in *S. aureus* by the MbcS pathway.

### Biochemical characterization of MbcS

**Figure 8:**
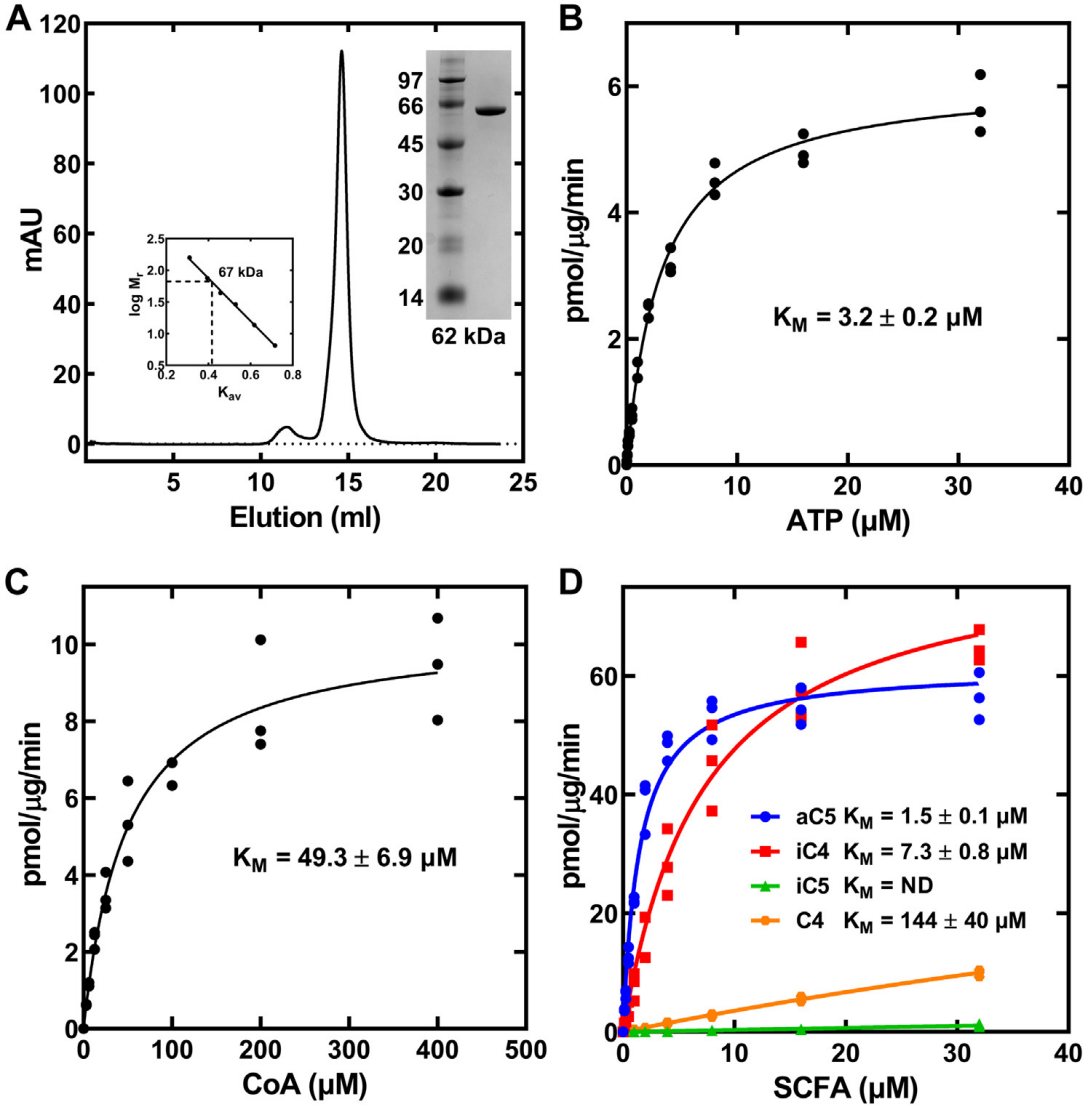
**Purification and biochemical properties of MbcS.***A*, MbcS was purified by Ni^2+-^affinity chromatography followed by gel filtration chromatography. *Left Inset*, MbcS is a typical globular monomer of 67 kDa based on the protein elution position on a calibrated gel filtration column. *Right Inset*, SDS gel electrophoresis illustrating the purity of the His-tagged MbcS. *B*, MbcS ATP K_M_ determined with 2.5 mM Mg^2+^, 200 μM CoA, and 50 μM aC5. *C*, MbcS CoA K_M_ determined in the presence of 2.5 mM ATP, 2.5 mM Mg^2+^, and 50 μM aC5. *D*, the K_M_ for each short-chain acid was determined in the presence of 2.5 mM ATP, 2.5 mM Mg^2+^, and 200 μM CoA. In all panels, data are from three independent experiments. The data were fit to a single site Michaelis–Menten equation using GraphPad software (https://www.graphpad.com/), and the fitted line with all the data points are shown. MbcS, methylbutyryl-CoA synthetase.

The *MbcS* gene was cloned into the pET28a vector and expressed in *Escherichia coli* as an amino-terminal His-tagged protein that was purified by Ni^2+^-affinity chromatography followed by gel filtration chromatography (Fig. 8*A*). MbcS was a monomer in solution based on its migration compared to reference proteins on a calibrated S-200 gel filtration column (Fig. 8*A*, *inset*). The homologous *Salmonella enterica* and *S. aureus* acetyl-CoA synthetases are also monomers (34, 35). The MbcS assay used LC-MS/MS to separate and detect the acyl-CoA product of the reaction (Fig. S5*A*). The amounts of acyl-CoA formed were quantified by comparing the areas of the acyl-CoA product peak to a standard curve generated using butyryl-CoA (Fig. S5*B*). The MbcS activity was linear with respect to input protein (Fig. S5*C*). Using this assay, the apparent K_M_ for ATP was 3.2 ± 0.2 μM (Fig. 8*B*) and the apparent K_M_ for CoA was 49.3 ± 6.9 μM (Fig. 8*C*). The most important biochemical characteristic of MbcS was its clear substrate selectivity (Fig. 8*D*). Although aC5 was the highest affinity substrate, both aC5 and iC4 had apparent K_M_’s <10 μM. Butyrate (C4) was a poor substrate (K_M_ = 144 μM) and iC5 was even worse (K_M_ > 500 μM). The main conclusion is that the substrate selectivity of MbcS *in vitro* (Fig. 8*D*) matches the selectivity for the incorporation of short-chain acids into PG *in vivo* (Figs. 5 and 6).

## Discussion

**Figure 9:**
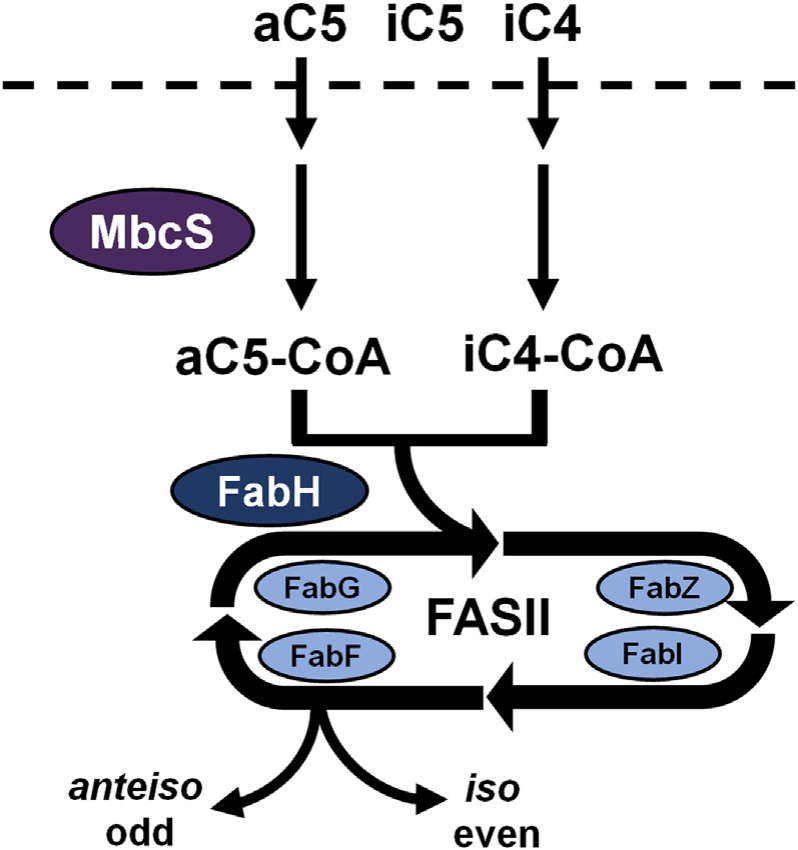
**Role of MbcS in activating 2-methylbutyrate (aC5) and isobutyrate (iC4) for the initiation of BCFA biosynthesis.** The MbcS pathway bypasses Bkd to support the formation of *anteiso* odd-chain and *iso* even-chain BCFA when aC5 and iC4 are present in the environment. Isovaleric acid (iC5) is a very poor substrate for MbcS meaning that the precursor to *iso* odd-chain fatty acids (iC5-CoA) is not produced in significant quantities from extracellular iC5.BCFA, branched-chain fatty acids; Bkd, branched-chain α-ketoacid dehydrogenase; MbcS, methylbutyryl-CoA synthetase.

The discovery of MbcS resolves the questions regarding the origin of BCFA in *S. aureus* strains lacking a functional Bkd. There is not a *de novo* biosynthetic bypass of Bkd, rather MbcS is positioned to salvage extracellular aC5 and iC4 by converting them to their respective CoA thioesters to directly provide substrates for FabH to initiate BCFA biosynthesis (Fig. 9). We find that Bkd-deficient strains cannot grow in chemically defined growth media unless it is supplemented with either aC5 or iC4 and that *mbcS* KO strains cannot incorporate short-chain acids present in the media into BCFA. The presence of MbcS accounts for the observation that strains lacking Bkd function are still able to produce BCFA when grown in rich undefined medias. This is due to the presence of substantial concentrations of short-chain acids present in these medias. It is not generally appreciated that short-chain acids are present in these undefined formulations and caution is advised when interpreting data with these medias because there are certainly other unknown components present that will influence the results of metabolic experiments. The amounts of short-chain acids varied significantly between the three different undefined rich medias analyzed here and other laboratories may find different results with these medias depending on the supplier and potentially the lot number. The BCFA requirement for *S. aureus* is also satisfied in Bkd-deficient strains by the addition of exogenous BCFA which can be incorporated into PG after their activation by fatty acid kinase (36, 37). BCFA are not found in sufficient concentrations in complex-rich medias to support the growth of Bkd-deficient strains (29, 38), although medias like BHI broth do contain sources of mammalian straight-chain fatty acids. The physiological role of MbcS in *S. aureus* metabolism remains to be experimentally determined. The fact that the MbcS substrate specificity matches the structures produced by the *de novo* pathway that supports branched-chain fatty acid synthesis (15) suggests MbcS has a role in maintaining these CoA thioester pools.

MbcS is an acyl-CoA synthetase with a high degree of relatedness to acetyl-CoA synthetases in *S. aureus* and *S. enterica*. Like these other synthetases, MbcS likely operates using a ping-pong mechanism to first form the aC5-AMP intermediate followed by the release of pyrophosphate. CoA then binds and is converted to aC5-CoA and AMP (39). The most notable biochemical property of MbcS is its substrate selectivity for aC5 and iC4 acids. Larger (iC5) and smaller (acetate and propionate) short-chain acids are poor substrates with the closely related C4 having an apparent K_M_ 20-fold higher than for iC4. This substrate selectivity of MbcS underlies the observed selectivity for the incorporation of extracellular short-chain acids into BCFA (Fig. 9). The specificity also matches the structures of the FabH precursors produced by the *de novo* BCAA biosynthetic pathway (iC4- and aC5-CoA) rather than the aC5- and iC5-CoA produced by the salvage BCAA pathway from extracellular Ile and Leu (15). The key residues defining the selectivity of acetyl-CoA synthetase are established (40) and although MbcS is predicted to adopt the same overall fold as acetyl-CoA synthetase, the structural basis for the exquisite substrate selectivity in MbcS will require determining crystal structures of ligand-bound MbcS. Bacterial acetyl-CoA synthetases are regulated by acetylation/deacetylation of a conserved lysine that acts as a switch that converts the active acetyl-CoA synthetase to an inactive enzyme with an acetylated lysine (35, 41, 42). Although MbcS has a lysine in the analogous position, we do not know whether MbcS is regulated by acetylation. This is a topic for future research but we think it unlikely that MbcS would be regulated by acetyl-CoA given its role in metabolism.

## Experimental procedures

### Materials

ATP, butyric acid, isobutyric acid, isovaleric acid, propionic acid, magnesium chloride, Tris base, and cadmium chloride were purchased from Sigma-Millipore. 2-Methylbutyric acid, acetic acid, chloroform, and methanol were purchased from Thermo Fisher Scientific. CoA and butyryl-CoA were purchased from Avanti. [U-^13^C]acetyl-CoA was from Sigma-Millipore. [^13^C_2_]Acetyl-ACP standard was made as described (15). [d7]Isobutyric acid and [d9]isovaleric acid were purchased from Cambridge Isotope Laboratories. BHI and tryptic soy broth bacterial media was purchased from BD Medical Technologies. LB was made using tryptone and yeast extract from Gibco and sodium chloride from Thermo Fisher Scientific. Defined media was made as described (15) using materials purchased from Sigma-Millipore and Thermo Fisher Scientific. Antibiotics and nickel resin were purchased from GoldBio. All analytical reagents were purchased from Sigma-Millipore or Thermo Fisher Scientific and are of HPLC grade or better. Bacterial strains and plasmids used in this study are listed in Tables S2 and S3.

### Lipid MS

Overnight cultures of strains JE2, NE1896, and NE1829 were diluted to A_60__0_ = 0.05 in LB with dimethyl sulfoxide or 1 mM aC5. After 5 h incubation at 37 °C cultures were washed twice with PBS and lipids were extracted using the Bligh and Dyer method (43). Lipid extracts were resuspended in chloroform/methanol (1:1). PG was analyzed using a Shimadzu Prominence Ultra Fast Liquid Chromatography (UFLC) system attached to a QTrap 4500 equipped with a Turbo V ion source (Sciex). Samples were injected onto an Acquity ultra performance liquid chromotography (UPLC) bridged ethylene hybrid hydrophilic interaction liquid chromotography, 1.7 um, 2.1 × 150 mm column (Waters) at 45 °C with a flow rate of 0.2 ml/min. Solvent A was acetonitrile, and solvent B was 15 mM ammonium formate, pH 3. The HPLC program was the following: starting solvent mixture of 96% A/4% B; 0 to 2 min, isocratic with 4% B; 2 to 20 min, linear gradient to 80% B; 20 to 23 min, isocratic with 80% B; 23 to 25 min, linear gradient to 4% B; 25 to 30 min, and isocratic with 4% B. The QTrap 4500 was operated in the Q1 negative mode. The ion source parameters for Q1 were as follows: ion spray voltage, −4500 V; curtain gas, 25 psi; temperature, 350 °C; ion source gas 1, 40 psi; ion source gas 2, 60 psi; and declustering potential, −40 V. The system was controlled by the Analyst software (Sciex, https://sciex.com/products/software/analyst-software). The sum of the areas under each peak in the mass spectra were calculated and the percentage of each molecular species present was calculated with LipidView software (Sciex, https://sciex.com/products/software/lipidview-software).

The samples were introduced to the QTrap 4500 by direct injection to perform product scans to verify the fatty acids present in a particular PG molecular species. The ion source parameters for negative mode product scan were as follows: ion spray voltage, −4500 V; curtain gas, 10 psi; collision gas, medium; temperature, 270 °C; ion source gas 1, 10 psi; ion source gas 2, 15 psi; declustering potential, −40 V; and collision energy, −50 V.

### Fatty acid analysis by GC

Fatty acid methyl esters were prepared from the JE2, NE1896, and NE1829 lipid extracts using anhydrous methanol/acetyl chloride. The fatty acid methyl esters were analyzed by a Hewlett–Packard model 5890 gas chromatograph equipped with a flame ionization detector and separated on 30 m × 0.536 mm × 0.50 μm DB-225 capillary column (Agilent). The injector was set at 250 °C and the detector was at 300 °C. The temperature program was as followed: initial temperature of 70 °C for 2 min, rate of 20 °C/min for 5 min (final 170 °C), rate of 2 °C/min for 10 min (final 190 °C), hold at 190 °C for 5 min, rate of 2 °C/min for 15 min (final 220°C), and hold at 220 °C for 5 min. The identity of fatty acid methyl esters was determined by comparing their retention times with fatty acid methyl ester standards (Matreya). The composition was expressed as weight percentages.

### Acyl-CoA measurement by MS

Overnight cultures of strains JE2, NE1896, and NE1829 were diluted to A_600_ = 0.05 in LB with dimethyl sulfoxide or 1 mM aC5. After 2 h incubation at 37 °C 10 ml culture was collected by centrifugation. The cell pellet was resuspended in 1 ml water, 2.4 ml methanol, and 1 ml chloroform and incubated at room temperature for 30 min. Chloroform 1.5 ml and 1.2 ml water were added to remove the lipids by phase separation. The aqueous top layer was collected and dried overnight in a Savant Speedvac Concentrator SPD 1010 (Thermo Fisher Scientific). The dried sample was resuspended in 300 μl 95% methanol + 15 mM ammonium hydroxide, 30 pmol of [^13^C_2_]acetyl-CoA (Millipore Sigma) was added and then 20 μl was injected into a mass spectrometer. Acyl-CoA was analyzed using a Shimadzu Prominence UFLC attached to a QTrap 4500 equipped with a Turbo V ion source (Sciex). Samples were injected onto an Acquity UPLC high strength silica (HSS) C18, 2.5 μm, 3.0 × 150 mm column at 40 °C (Waters) using a flow rate of 0.4 ml/min. Solvent A was 100 mM ammonium formate pH 5 + 2% acetonitrile + 0.1% tributylamine (TBA), and Solvent B was 95% acetonitrile + 10 mM ammonium formate pH 6.3 + 0.1% TBA. The HPLC program was the following: starting solvent mixture of 100% A/0% B, 0 to 2 min isocratic with 0% B; 2 to 20 min linear gradient to 50% B; 20 to 24 min linear gradient to 95% B; 24 to 29 min isocratic with 95% B; 29 to 30 min linear gradient to 0% B; and 30 to 35 min isocratic with 0% B. The QTrap 4500 was operated in the positive mode, and the ion source parameters were as follows: ion spray voltage, 5500 V; curtain gas, 30 psi; temperature, 450 °C; collision gas, medium; ion source gas 1, 25 psi; ion source gas 2, 30 psi; declustering potential, 60 V; and collision energy, 45 V. The multiple reaction monitoring (MRM) transitions were as follows: CoA, 768.1/261.1; C2-CoA, 810.1/303.1; C3-CoA, 824.1/317.1; *i*C4- and C4-CoA, 838.1/331.1; *i*C5- and *a*C5-CoA, 852.1/345.1; Mal-CoA, 854.1/347.1; (+9)*i*C5-CoA, 861.1/354.1; (+7)*i*C4-CoA, 845.1/338.1; and [^13^C_2_]acetyl-CoA, 812.1/305.1. [^13^C_2_]Acetyl-CoA was used as the internal standard. The system was controlled by the Analyst software (Sciex, https://sciex.com/products/software/analyst-software) and analyzed with MultiQuant 3.0.2 software (Sciex, https://sciex.com/products/software/multiquant-software). Peaks corresponding to individual acyl-CoA species were quantified relative to the internal standard.

### Acyl-ACP measurement by MS

Overnight cultures of strains JE2, NE1896, and NE1829 were diluted to A_600_ = 0.05 in LB with DMSO or 1 mM aC5. After 2 h incubation at 37 °C samples were prepared for acyl-ACP quantification as described (44). Briefly, 1 ml aliquots of cultures were removed to a 1.5 ml Eppendorf tube containing 250 μl ice cold 10% trichloroacetic acid. The tubes were inverted and kept on ice. Harvested cells were washed with acetone, dried, and then stored at −80 °C until further processing. Pellets were resuspended in 100 μl of lysis buffer (50 mM sodium phosphate buffer, pH 7.2, 1 mM ascorbic acid, 2 mM ethylenediaminetetraacetic acid, 6 M urea). Lysis buffer was prepared just prior to use. [^13^C_2_]Acetyl-ACP was added as an internal standard (1 μl of 0.0125 μg/μl [^13^C_2_]acetyl-ACP). A chloroform/methanol extraction was performed by adding 400 μl methanol, followed by 100 μl of chloroform. Samples were sonicated in a sonication bath (Thermo Fisher Scientific CPXH Series 1.9L) for 10 min at room temperature to resuspend. Phases were separated by adding 300 μl 200 mM formate buffer, pH 3.9. The upper phase was discarded and 300 μl of methanol was added to remaining sample. Precipitated proteins were washed with 300 μl of methanol and then dried. ACP was resuspended in 10 μl of 100 mM sodium phosphate buffer, pH 6.5 and sonicated for 10 min at room temperature in an ultrasonic bath. Insoluble debris was pelleted and 5 μl of resuspended ACP was removed to a clean tube. The sample was digested by adding 0.5 μg endoproteinase Asp-N (Sigma-Millipore) in 10 μl of 100 mM Tris, pH 7.5. Samples were incubated 1 h at room temperature to allow complete digestion with minimal degradation of acyl chains. Proteolysis was quenched with 15 μl methanol.

Acyl-ACP species were analyzed using a Shimadzu Prominence UFLC attached to a QTrap 4500 equipped with a Turbo V ion source (Sciex). Samples were injected onto an Acquity UPLC Discovery B10 Wide Pore C18, 3 μm, 2.1 × 100 mm column (Millipore Sigma) at 25 °C with a flow rate of 0.2 ml/min. Solvent A was 10 mM ammonium formate, pH 3.6, in 10% acetonitrile, and solvent B was 10 mM ammonium formate, pH 3.6, in 90% acetonitrile. The HPLC program was the following: starting solvent mixture of 100% A/0% B; 0 to 4 min, linear gradient to 10% B; 4 to 12 min, linear gradient to 100% B; 12 to 17 min, isocratic with 100% B; 17 to 20 min, linear gradient to 0% B; and 20 to 25 min, isocratic with 0% B. The QTrap 4500 was operated in the positive mode, and the ion source parameters were as follows: ion spray voltage, 5500 V; curtain gas, 30 psi; temperature, 400 °C; collision gas, medium; ion source gas 1, 25 psi; and ion source gas 2, 30 psi. The MRM transitions were as exactly described (15). The system was controlled by the Analyst software (Sciex) and analyzed with MultiQuant 3.0.2 software (Sciex). The MRM transitions were exactly as described (15), except for the (+7)even-chain acyl-ACPs and the (+9)odd-chain acyl-ACPs, which either 7 or 9 was added to both the Q1 and Q3 MRM masses described for normal acyl-ACPs (15). [^13^C_2_]Acetyl-ACP was used as the internal standard. Peaks corresponding to individual acyl-ACP species were quantified relative to the internal standard.

### Short-chain fatty acid quantification

Short-chain fatty acid analysis was performed as described (45, 46). Briefly, 40 μl of media, 20 μl of 200 mM 3-nitrophenylhydrazine-HCl in 50% acetonitrile, 20 μl of 120 mM 1-ethyl-3-(3-dimethylaminopropyl)carbodiimide-HCl in 6% pyridine and 50% acetonitrile, and 5 μl of 5 mM [d9]isovalerate were added to a glass vial. The mixture was vortexed and incubated at 40 °C for 30 min. Samples were cooled on ice for 1 min and diluted with 915 μl of 10% acetonitrile. Samples were analyzed by a Shimadzu Prominence UFLC attached to a QTrap 4500 equipped with a Turbo V ion source (Sciex). Samples were injected onto an Acquity UPLC HSS C18, 2.5 μm, 3.0 × 150 mm column at 45 °C (Waters) using a flow rate of 0.4 ml/min. Solvent A was water + 0.1% formic acid, and Solvent B was acetonitrile + 0.1% formic acid. The HPLC program was the following: starting solvent mixture of 85% A/15% B, 0 to 2 min isocratic with 15% B; 2 to 12 min linear gradient to 55% B; 12 to 16 min linear gradient to 100% B; 16 to 23 min isocratic with 100% B; 23 to 25 min linear gradient to 15% B; and 25 to 30 min isocratic with 15% B. The QTrap 4500 was operated in the negative mode, and the ion source parameters were as follows: ion spray voltage, −4500 V; curtain gas, 30 psi; temperature, 400 °C; collision gas, medium; ion source gas 1, 15 psi; ion source gas 2, 20 psi; declustering potential, −70 V; and collision energy, −27 V. The MRM transitions are as follows: acetate (C2), 194.1/136.9; propionate (C3), 208.1/136.9; isobutyrate (iC4) and butyrate (C4), 222.1/136.9; 2-methyl-butyrate (aC5), isovalerate (iC5), and valerate (C5), 236.1/136.9; malonate (Mal), 373.1/178.0; and [d9]isovalerate, 245.1/136.9. [d9]Isovalerate was used as the internal standard. The system was controlled by the Analyst software (Sciex) and analyzed with MultiQuant 3.0.2 software (Sciex). Peaks corresponding to individual short-chain fatty acid species were quantified relative to the internal standard.

### aC5/iC4 auxotrophy

Overnight cultures of strain NE1896 grown in defined media with 250 μM aC5 were washed twice with defined media with no supplement at room temperature. Cells were resuspended and passed through a 40-μm filter to remove clumped cells. Cultures were diluted to A_600_ = 0.05 in defined media with water or 100 μM each of acetate (C2), butyrate (C4), isobutyrate (iC4), 2-methylbutyrate (aC5), or isovalerate (iC5). After 7 h incubation at 37 °C, the A_600_ was measured.

### Identification of the MbcS gene

KOs for candidate genes for the synthetase were pulled out of the NARSA collection and screened. Overnight cultures were grown in defined media without Ile and Leu with 50 μM aC5 for 4 to 5 h and lipids were extracted as described above. The ratio of the 32-carbon peak area to the 35-carbon peak area was used to assess whether or not the aC5 was being incorporated into PG. A dose-response curve for aC5 was conducted using the same experimental parameters and varying the amount of aC5 added to strain JE2.

The *mbcS* complemented strain was made by putting the *mbcS* sequence into vector pPJ657, which is the pCN51 vector with a chloramphenicol resistance marker replacing the kanamycin resistance marker. The resulting plasmid, pPJ658, has the *mbcS* gene under the control of a cadmium inducible promotor. pPJ658 was transformed into strain NE1036. The complement strain was grown as above in defined media without Ile or Leu, with 50 μM aC5 and 2.5 μM cadmium chloride. Lipids were extracted as above and the 32/35-PG ratio was calculated.

### Incorporation of extracellular short-chain acids

Overnight cultures of JE2 were cut back to A_600_ = 0.05 in defined media without Ile and Leu with 25 μM [d7]isobutyrate plus 25 μM [d9]isovalerate. Cells were grown at 37 °C for 4.5 h and lipids were extracted from 10 ml culture samples as described above. For the CoA and ACP heavy acid incorporation experiments, cells were grown for 4 h at 37 °C in defined media without Ile and Leu the culture was split and 50 μM [d7]isobutyrate and 50 μM [d9]isovalerate were added to one culture. After 30 min at 37 °C, 1 ml aliquots were removed for ACP sample processing and 10 ml aliquots were removed for CoA sample processing as described above.

### MbcS expression and purification

The MbcS (*SAUSA300_2542*) sequence from *S. aureus* was amplified from genomic DNA and cloned by Gibson Assembly into pET28a under control of an IPTG inducible promotor to create plasmid pPJ656. Protein was expressed using pPJ656 in *E. coli* BL21(DE3) cells. Protein expression was induced with IPTG overnight at 16 °C. After cell lysis, protein was purified using nickel affinity chromatography. The pooled purified protein was dialyzed overnight into 20 mM Tris pH 7.5 with 200 mM NaCl and further purified by size exclusion chromatography. MbcS was concentrated using Amicon Ultra centrifugal filters and quantified by UV absorption.

### MbcS activity assay

Synthetase activity of MbcS was assayed in a reaction mixture containing 0.2 mM Tris (pH 7.5), with varying concentrations of MgCl_2_, ATP, CoA, short-chain fatty acid, and enzyme as indicated in individual figures. Reactions were initiated by the addition of MbcS. After incubation at 37 °C for 15 min, the reaction mixtures were placed on ice and methanol was added at 1:1 ratio by volume to stop the reaction. Experiments were performed in triplicate. Reactions were analyzed by a Shimadzu Prominence UFLC attached to a QTrap 4500 equipped with a Turbo V ion source (Sciex). Samples were injected onto an Acquity UPLC HSS C18, 2.5 μm, 3.0 × 150 mm column at 40 °C (Waters) using a flow rate of 0.4 ml/min. Solvent A was 100 mM ammonium formate pH 5 + 2% acetonitrile + 0.1% TBA, and Solvent B was 95% acetonitrile + 10 mM ammonium formate pH 6.3 + 0.1% TBA. The HPLC program was the following: starting solvent mixture of 100% A/0% B; 0 to 2 min isocratic with 0% B; 2 to 12 min linear gradient to 100% B; 12 to 16 min isocratic with 100% B; 16 to 17 min linear gradient to 0% B; and 17 to 20 min isocratic with 0% B. The QTrap 4500 was operated in the positive mode, and the ion source parameters were as follows: ion spray voltage, 5500 V; curtain gas, 30 psi; temperature, 450°C; collision gas, medium; ion source gas 1, 25 psi; ion source gas 2, 30 psi; declustering potential, 60 V; and collision energy, 40 V. The MRM transitions were as follows: CoA, 768.1/261.1; C2-CoA, 810.1/303.1; C3-CoA, 824.1/317.1; iC4- and C4-CoA, 838.1/331.1; iC5- and aC5-CoA, 852.1/345.1; malonyl-CoA, 854.1/347.1; succinyl- and methylmalonyl-CoA, 868.1/361.1; and fumaryl-CoA, 866.1/359.1. The system was controlled by the Analyst software (Sciex) and analyzed with MultiQuant 3.0.2 software (Sciex). Substrate and product were separated (Fig. S5*A*) and the amounts of the individual CoA species were quantified based on a C4-CoA standard curve (Fig. S5*B*). MbcS activity was linear with protein concentration (Fig. S5*C*).

## Data availability

All the data produced for this work are contained within the article.

## Supporting information

This article contains supporting information (26, 47).

## Conflict of interest

The authors declare that they have no conflicts of interest with the contents of this article.
