# Supplementary material for: A short-chain acyl-CoA synthetase that supports branched-chain fatty acid synthesis in Staphylococcus aureus
Source: J Biol Chem. 2023 Feb 16;299(4):103036. doi: 10.1016/j.jbc.2023.103036 (PMC10026030; doi:10.1016/j.jbc.2023.103036)
Supplement: Supplemental Tables S1–S3 and Figures S1–S5 [file mmc1.pdf]

# Supplemental Materials

## A short-chain acyl-CoA synthetase that supports branched-chain fatty acid synthesis in *Staphylococcus aureus*

Sarah G. Whaley, Matthew W. Frank, and Charles O. Rock

**Tables S1-S3.**
**Figures S1-S5.**

**Table S1**
**Fatty acid compositions of strains JE2 (wild-type) and NE1896 (*lpdA*::ϕNΣ).**

| Fatty acid[a] | Strain | | |
|---|---|---|---|
| | **JE2[b]** | **NE1896[b]** | **NE1896[c]** |
| *iso* 14:0 | 0.6 ± 0.2 | 5.2 ± 1.2 | 0.9 ± 0.4 |
| 14:0 | ND[d] | ND | 0.1 ± 0.1 |
| *iso* 15:0 | 6.4 ± 1.0 | ND | ND |
| *anteiso* 15:0 | 26.8 ± 1.2 | 17.7 ± 3.4 | 24.2 ± 2.3 |
| 15:0 | ND | ND | ND |
| *iso* 16:0 | 2.3 ± 0.2 | 12.8 ± 1.9 | 3.4 ± 0.6 |
| 16:0 | 1.3 ± 0.1 | 2.6 ± 0.5 | 2.2 ± 0.4 |
| *iso* 17:0 | 7.4 ± 0.0 | ND | ND |
| *anteiso* 17:0 | 27.9 ± 0.7 | 4.1 ± 0.6 | 18.9 ± 0.2 |
| 17:0 | ND | 0.9 ± 0.2 | 0.4 ± 0.1 |
| *iso* 18:0 | 1.7 ± 0.1 | 8.5 ± 0.5 | 3.0 ± 0.1 |
| 18:0 | 7.5 ± 1.2 | 20.1 ± 2.0 | 16.4 ± 2.3 |
| *iso* 19:0 | 3.6 ± 0.8 | ND | ND |
| *anteiso* 19:0 | 10.0 ± 1.9 | 1.6 ± 0.5 | 8.5 ± 1.6 |
| 19:0 | 0.4 ± 0.1 | 2.9 ± 0.4 | 2.5 ± 0.1 |
| *iso* 20:0 | 0.4 ± 0.1 | 2.9 ± 0.7 | 1.0 ± 0.2 |
| 20:0 | 3.6 ± 0.6 | 20.7 ± 3.9 | 18.4 ± 1.0 |

[a]Fatty acid composition is in weight percent.
[b]Strains JE2 and NE1896 were grown in LB
[c]Strain NE1896 was grown in LB supplemented with 1 mM aC5.
[d]ND means not detected.

**Table S2**
**Bacterial strains used in this study**.

| Strain | Genotype | Gene name | Description | Source |
|---|---|---|---|---|
| JE2 | | | parent wild-type strain | Fey, *et al.*[a] |
| NE1829 | JE2 SAUSA300_1465::ϕNΣ | *bkdA2* | branched-chain alpha-keto acid dehydrogenase E1 | Fey, *et al.*[a] |
| NE1896 | JE2 SAUSA300_1467::ϕNΣ | *lpdA* | dihydrolipoamide dehydrogenase | Fey, *et al.*[a] |
| NE162 | JE2 SAUSA300_0559::ϕNΣ | | putative CoA ligase | Fey, *et al.*[a] |
| NE385 | JE2 SAUSA300_1679::ϕNΣ | *acsA* | acetyl-CoA synthetase | Fey, *et al.*[a] |
| NE539 | JE2 SAUSA300_0228::ϕNΣ | *fadE* | acyl-CoA synthetase | Fey, *et al.*[a] |
| NE1036 | JE2 SAUSA300_2542::ϕNΣ | *mbcS* | putative acetate-CoA ligase | Fey, *et al.*[a] |
| SW658 | NE1036/pMbcS (pPJ658) | | *mbcS* complement strain | This study |

[a]Fey, P. D., Endres, J. L., Yajjala, V. K., Widhelm, T. J., Boissy, R. J., Bose, J. L., and Bayles, K. W. (2013) A genetic resource for rapid and comprehensive phenotype screening of nonessential *Staphylococcus aureus* genes. *MBio* **4**, e00537-00512

**Table S3**
**Plasmids used in this study.**

| Plasmid | Description | Source |
|---|---|---|
| pET-28a | IPTG inducible protein expression in *E. coli* | Novagen |
| pPJ656 | *mbcS* in pET-28a | This study |
| pCN51 | Low copy number, cadmium inducible expression vector | Charpentier, *et al.*[a] |
| pPJ657 | pCN51 vector with chloramphenicol selection | This study |
| pMbcS | *mbcS* in pPJ658 | This study |

[a]Charpentier, E., Anton, A. I., Barry, P., Alfonso, B., Fang, Y., and Novick, R. P. (2004) Novel cassette-based shuttle vector system for gram-positive bacteria. *Appl. Environ. Microbiol* **70**, 6076-6085

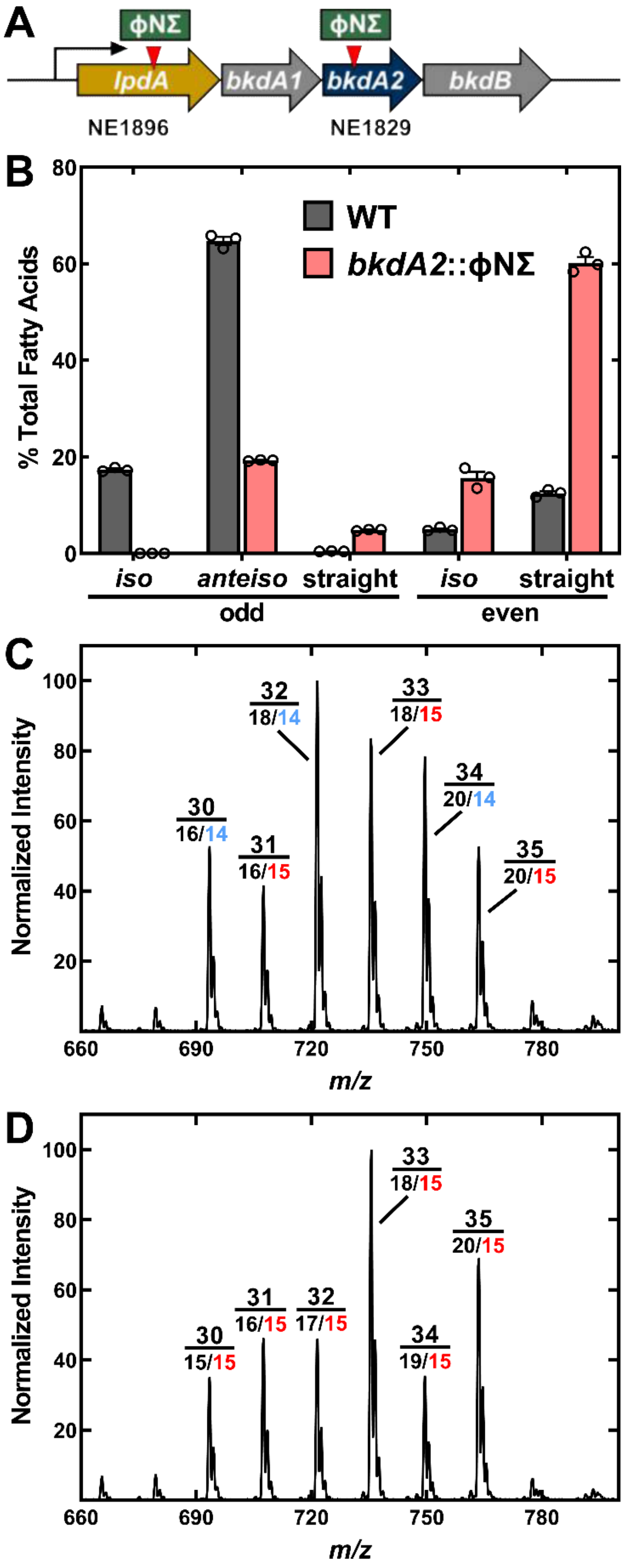


**Figure S1. The *S. aureus bkd* operon and lipidomic characteristics of strain NE1829 (*bkdA2*::ϕNΣ).** *A*, diagram of the 4-gene *bkd* operon showing the locations of the ϕNΣ transposon insertions in the *lpdA* and *bkdA2* genes (red triangles). *B*, fatty acid composition of strain NE1829 (*bkdA2*::ϕNΣ) compared to the wild-type parent strain JE2 grown in LB. The strain JE2 data are the same as in Fig. 2*A* for comparison. *C*, PG molecular species in strain NE1829 (*bkdA2*::ϕNΣ) grown in LB. *D*, PG molecular species of strain NE1829 (*bkdA2*::ϕNΣ) grown in LB supplemented with 1 mM aC5.

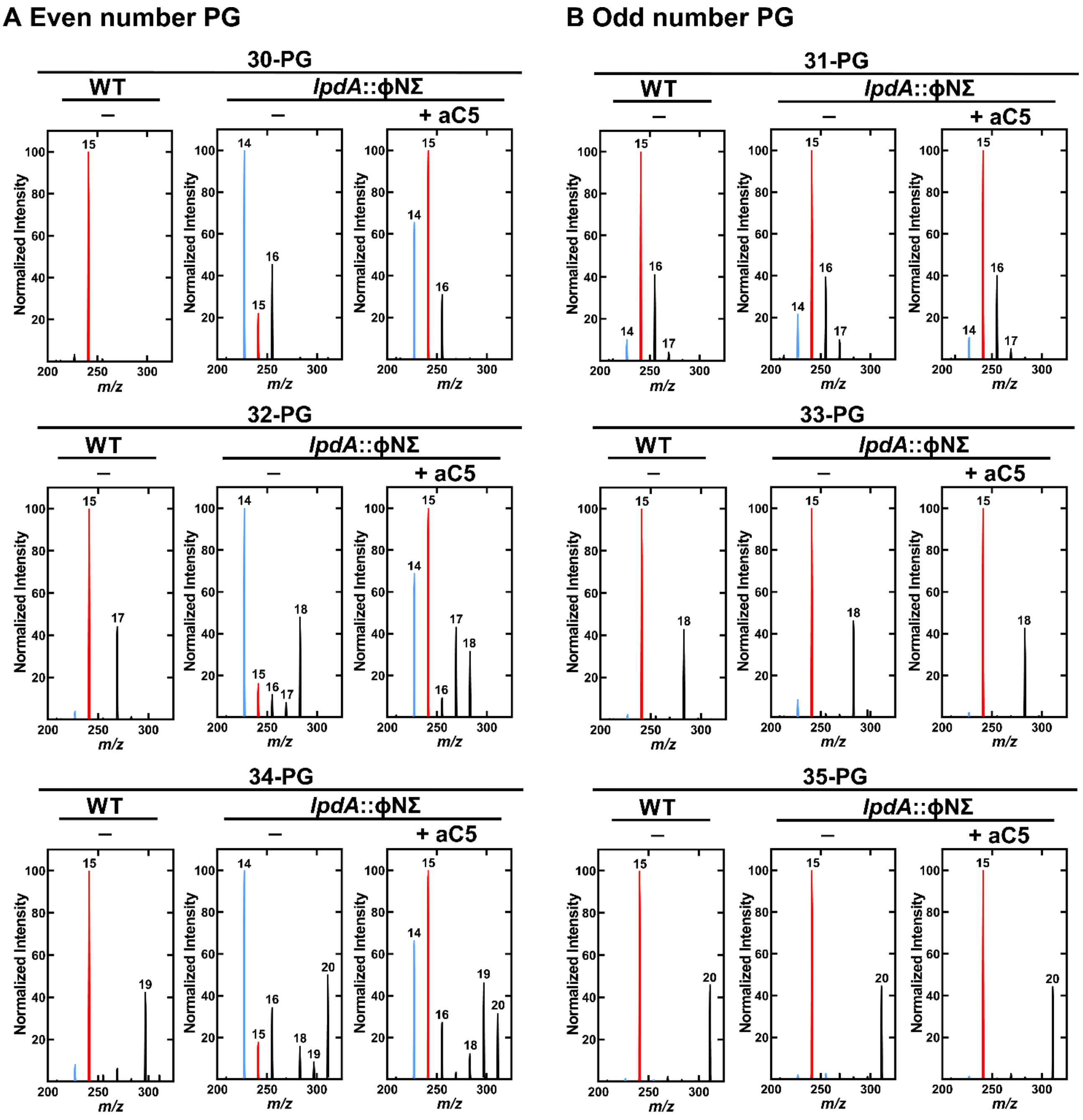


**Figure S2. Fragmentation of the PG molecular species in strain NE1896 (*lpdA*::ϕNΣ) grown in LB with and without a 1 mM aC5 supplement.** The individual PG peaks in the mass spectra shown in Figs. 2*B*, 2*C* and 2*D* were fragmented to determine the identity and positional distribution of the acyl chains in each PG peak. The 2-position acyl chain is characteristically ~2 times more intense the 1-position acyl chain (see text). Identity of the BCFA chains is deduced based on knowledge of the fatty acid composition (Table S1). The i14 acyl chain is cyan and the a15 acyl chain is red.

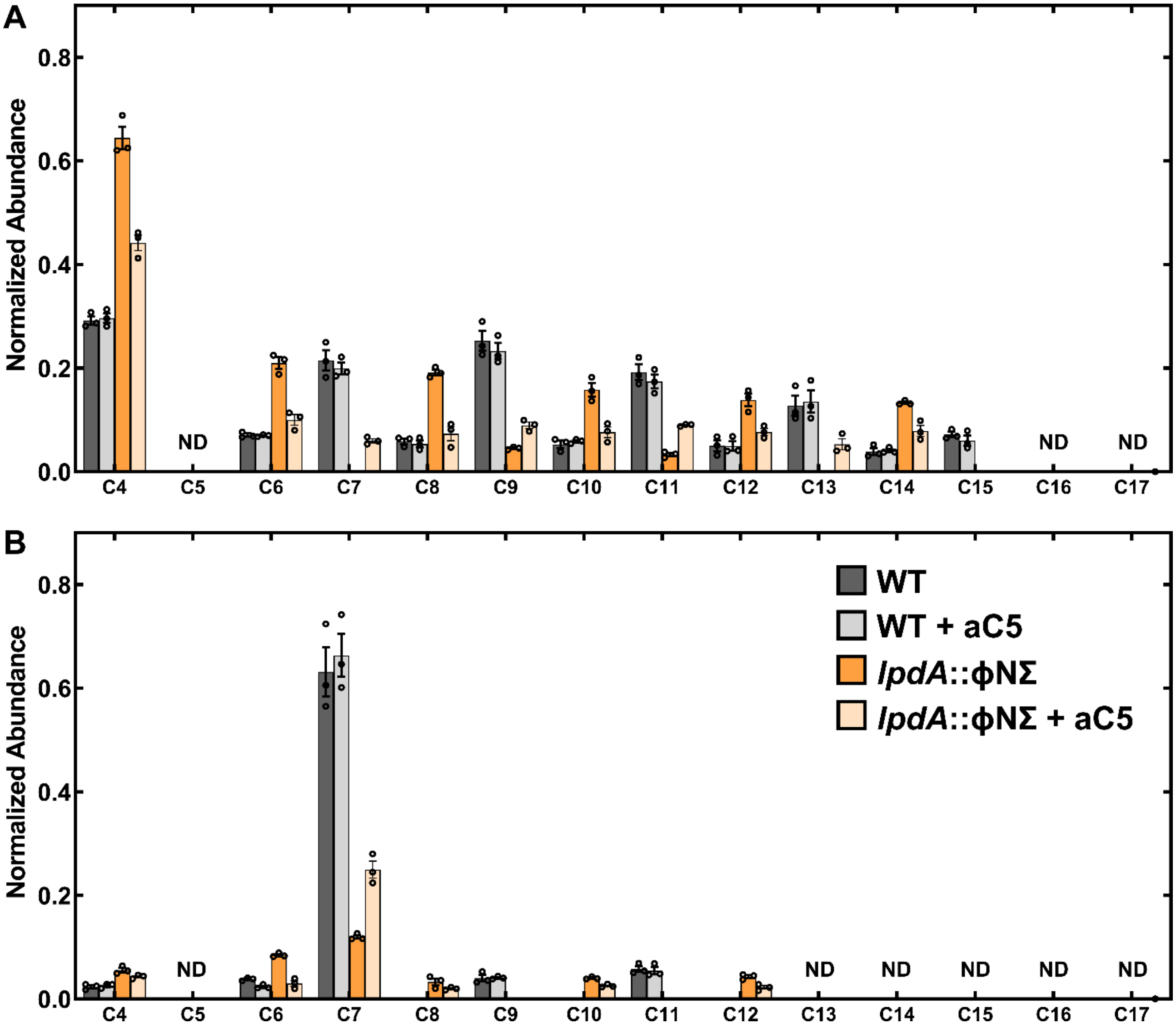


**Figure S3. Impact of aC5 supplementation on the composition of the 3-hydroxy- and *trans*-2-acyl-ACP pools.** Strains JE2 (wild-type) and NE1896 (*lpdA*::ϕNΣ) were grown in LB or LB supplemented with 1 mM aC5. The cells were collected by acid precipitation and protease digested. The FASII 3-hydroxy- and *trans*-2-acyl-ACP intermediates were detected by LC-MS/MS and the relative abundance of each peak was calculated based on the [$^{13}C_2$]acetyl-ACP standard. *A*, the distribution of 3-hydroxy-acyl-ACP chain lengths. *B*, the distribution of *trans*-2-acyl-ACP chain lengths. *Inset*, the color key for both panels.

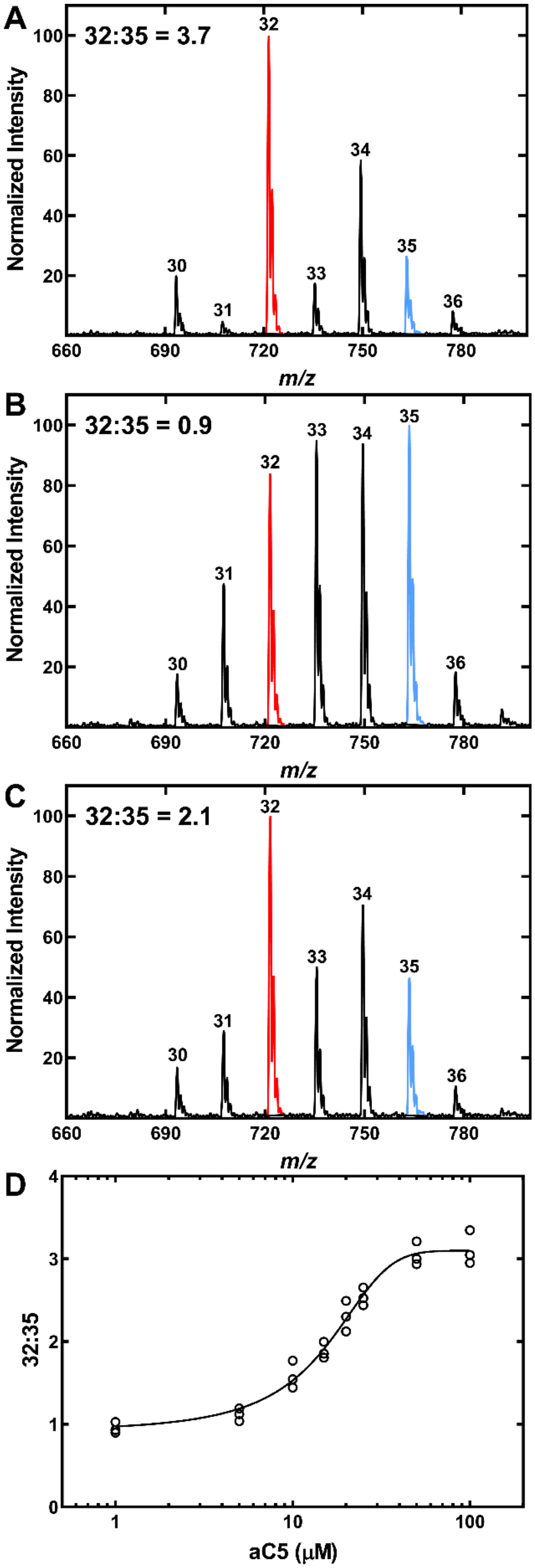


**Figure S4. The impact of aC5 supplementation on the PG molecular species in wild-type strain JE2.** *A*, strain JE2 grown in defined medium had a 32:35 PG ratio of 3.7. *B*, strain JE2 grown in defined medium lacking Ile and Leu had a 32:35-PG ratio of 0.9. *C*, strain JE2 grown in defined medium lacking Ile and Leu supplemented with 500 μM aC5 had a 32:35-PG ratio of 2.1. *D*, the 32:35-PG ratio in strain JE2 grown in defined medium lacking Ile and Leu plotted as a function of the aC5 concentration in the media.

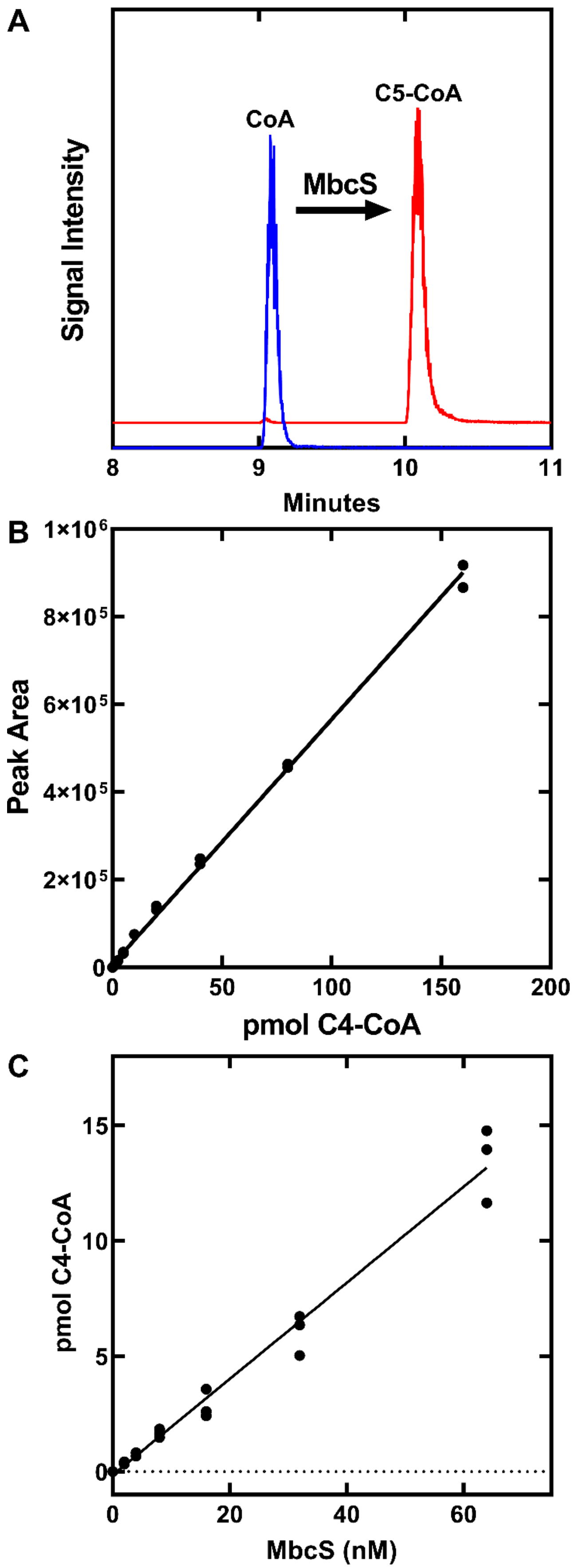


**Figure S5. Assay for MbcS.** MbcS assays were performed as described in Experimental Procedures and the reactions stopped by the addition of methanol. *A*, in this example, the CoA substrate (blue) was separated from the aC5-CoA product (red), and the peak areas analyzed with MultiQuant™ 3.0.2 software (Sciex). *B*, the C4-CoA calibration curve used to calculate the pmoles of acyl-CoA product formed in the MbcS reactions. *C*, the MbcS assay was linear with protein concentration. This example used 2-methylbutyric acid as the substrate.
